# Sustainable Development and Polymer-Based Functional Innovation in the Lacquer Industry: Resources, Technologies, and Industrialization Pathways

**DOI:** 10.3390/polym18131578

**Published:** 2026-06-25

**Authors:** Yihua Qian, Xiaoyu Wu, Yujia Liu, Xinhao Feng, Xinyou Liu

**Affiliations:** 1 College of Furnishing and Industrial Design, Nanjing Forestry University, Nanjing 210037, China; 2411404109qyh@njfu.edu.cn (Y.Q.); xiaoyv@njfu.edu.cn (X.W.); liuyujia@njfu.edu.cn (Y.L.); fengxinhao@njfu.edu.cn (X.F.); 2 Co-Innovation Center of Efficient Processing and Utilization of Forest Resources, Nanjing Forestry University, Nanjing 210037, China

**Keywords:** lacquer industry, lacquer resources, urushiol, natural lacquer, bio-based coatings, laccase curing, functional coatings, lacquer polymerization

## Abstract

Natural lacquer, a bio-based polymer derived from Toxicodendron vernicifluum, has attracted renewed scientific interest as a sustainable coating material with exceptional mechanical durability, chemical resistance, and aesthetic qualities. This review synthesizes current knowledge on the chemical composition, enzymatic curing mechanisms, and structure–property relationships of lacquer-based polymer systems, with particular focus on recent advances in functional modification and processing technology. Key findings indicate that laccase-catalyzed oxidative polymerization, operating optimally at pH 6.0–7.5 and 20–30 °C, governs the formation of a highly cross-linked urushiol network whose properties are fundamentally determined by side-chain unsaturation and emulsion stability. Mechanistic analysis reveals that polyurethane hybridization improves weathering resistance by introducing flexible aliphatic segments and additional hydrogen-bonding cross-links, while graphene oxide incorporation enhances anticorrosion performance through a physical barrier mechanism that prolongs ionic diffusion pathways. UV-curable LPEA derivatives achieve an 83% reduction in curing time relative to ambient-cured lacquer, enabling integration with industrial spray-coating lines. Despite these advances, several critical limitations remain inadequately resolved. Allergen reduction strategies have not yet achieved sufficient quantitative efficiency for large-scale commercial deployment, and the long-term stability of nanocomposite lacquer films under sustained UV exposure and hydrothermal conditions is not well established. Furthermore, most high-performance modification systems reported in the literature are demonstrated only on laboratory scale, with scalability, substrate compatibility, and lifecycle performance remaining largely unvalidated. The review identifies the absence of standardized performance evaluation protocols and the fragmentation of structure–property data across studies as key barriers to systematic progress, and proposes that future work prioritize the development of integrated processing–modification–performance frameworks to guide the rational design of next-generation lacquer-based functional materials.

## 1. Introduction

At present, China’s lacquer industry is at a turning point, facing the dual challenge of inheritance and innovation while being influenced by national intangible cultural heritage policies, rural revitalization, and ecological civilization construction [1]. The national promotion of creative transformation and innovative development of traditional culture, along with growing public recognition of cultural and ecological consumption, has created favorable social conditions and market demand for the transition from “niche skills” to industrial advantages [2]. Meanwhile, advances in materials science, intelligent production, and digital technologies are transforming production processes, product styles, and design possibilities, shifting lacquer from workshop-based handicraft to an industrial form integrating new technologies, designs, and brands [3,4].

Despite this potential, structural contradictions limit sustainable development. First, resource constraints are intensifying [5]; the lacquer tree, the industry’s core raw material, suffers from reduced cultivation areas due to low economic returns [6], slow development of superior varieties, large-scale planting, and pest control [7], and traditional harvesting methods overlook resource protection, resulting in insufficient supply, rising prices, and unstable quality. Second, a shortage of professional talent exists. Traditional artisan education is limited in scale and scope, failing to meet contemporary needs for integrated creative, technological, and marketing skills, leading to slow innovation, product iteration, and industry momentum. Third, industrialization and marketization are insufficient. Lacquer products have low acceptance, often imitate ancient designs, and fail to meet modern aesthetic and functional demands [8,9]. Fragmented production, low conversion of research achievements, and lack of cultural value refinement further hinder development.

Therefore, addressing the contradictions in resources, technology, and market, and constructing a sustainable, innovation-driven, and market-oriented industrial ecosystem is urgent. This paper provides theoretical references and practical strategies to overcome current bottlenecks, optimize and upgrade the industrial chain, and achieve coordinated cultural inheritance and industrial revitalization through a comprehensive review of resource management, key technological innovations, and industrialization models [10].

## 2. The History of Lacquers in China

### 2.1. Origins and Early Development: From the Neolithic Period to the Zhou Dynasty

The history of lacquer use in China is among the longest of any craft tradition in the world, with archaeological evidence placing its origins in the Neolithic period approximately 8000 years ago. The earliest known lacquer artifact is a mulberry-wood lacquered bow unearthed in 2002 at the Kuahuqiao site in Hangzhou, Zhejiang Province. This discovery, confirmed through enzyme-linked immunosorbent assay (ELISA) analysis, demonstrates that the inhabitants of the Yangtze River Delta had already mastered the fundamental technique of applying natural lacquer sap to wooden substrates [11,12]. A cinnabar-lacquered wooden bowl recovered from the Hemudu site in Yuyao, Zhejiang, dating to approximately 7000 years ago, further confirms that lacquer was not merely a functional waterproofing agent but had already acquired aesthetic significance in everyday life.

During the Shang Dynasty (c. 1600–1046 BCE), lacquerware production became increasingly sophisticated in both technique and decoration. Ornamental motifs such as the taotie mask closely echoed the aesthetic vocabulary of contemporaneous bronze vessels, while inlay techniques incorporating turquoise and shell began to appear. By the Western Zhou and Spring and Autumn periods, the use of lacquerware had gradually expanded beyond ritual and elite contexts into broader social settings. It was during the Warring States period (475–221 BCE), centered on the state of Chu in present-day Hunan and Hubei provinces, that lacquerware production reached an early zenith. Chu lacquerware from this era encompassed a wide range of object types including furniture, musical instruments, and ceremonial vessels, with polychrome painting and inlay techniques having attained a high degree of maturity [13,14]. The scale of production had grown to the point where dedicated official positions were established to oversee lacquer tree cultivation and lacquerware manufacturing, marking a significant transition from craft activity to organized industry.

### 2.2. Classical Flourishing: The Qin–Han Peak and the Song–Yuan Period

The Qin unification and the subsequent Han Dynasty (206 BCE–220 CE) represent the first major apex of China’s lacquer tradition. State-supervised official workshops produced lacquerware of exceptional quality and considerable volume for the imperial household and the aristocracy. The several hundred well-preserved lacquer objects excavated from the Mawangdui Han tombs in Changsha, Hunan in 1972—including food vessels, cosmetic boxes, and musical instruments—provide the most vivid material testament to this flourishing period. The fluid polychrome brushwork and refined black-ground painting on these objects demonstrate that Han craftsmen had achieved comprehensive mastery of the full production sequence, from base preparation and successive lacquer coating to surface decoration and polishing [15]. Han-period lacquerware functioned simultaneously as utilitarian daily ware, ritual object, and marker of social prestige, reflecting the deep integration of lacquer culture into the fabric of Han society.

During the Tang Dynasty (618–907 CE), a number of technically refined decorative methods emerged, most notably jinyinpingtuo, in which gold and silver foil cut into intricate designs was inlaid flush with the lacquer surface, and luodian mother-of-pearl inlay, both of which expressed the Tang court’s taste for luxury and cosmopolitan splendor. The Song Dynasty (960–1279 CE) saw a stylistic shift toward restraint and understatement, with monochromatic lacquerware and spare vessel forms gaining favor. At the same time, the art of carved lacquer (diaoqi) rose to prominence, and techniques such as tihong (carved red lacquer) and tiqie (carved rhinoceros-pattern lacquer) laid the foundation for the high-relief carved lacquer traditions that would fully flourish in the Ming and Qing periods.

### 2.3. Peak Achievement, Modern Transformation, and Contemporary Development

The Ming (1368–1644) and Qing (1644–1912) dynasties are generally regarded as the second, and thus far the highest, developmental peak of Chinese lacquer art, with the breadth and precision of craftsmanship reaching unprecedented levels. Ming dynasty literature records over 14 major categories and nearly 400 varieties of lacquerworking techniques. Masters of carved lacquer such as Zhang Cheng and Yang Mao gained historical renown; regional schools such as Fuzhou’s bodiless lacquerware and Pingyao’s polished-glare lacquerware each possessed distinct characteristics and continue to this day. Lacquerware supervised and manufactured by the Qing imperial workshops comprehensively integrated various techniques, including carving, inlay, painting, and filling, reaching the ultimate level in both conceptual design and technical precision [16].

Since the 20th century, the lacquer art tradition has faced severe challenges, including the collapse of the patronage system, gaps in skills transmission, and the impact of industrial materials. The lacquer painting art that emerged in the 1980s elevated lacquer art from the realm of arts and crafts to that of pure art, fostering an active dialogue with contemporary visual culture [17]. In recent years, lacquer art has entered a new stage of multidisciplinary integration. Practitioners are combining traditional techniques with modern materials science, digital design tools, additive manufacturing technology, and cross-border collaborations with other artistic media such as ceramics and metal, exploring entirely new forms of artistic expression.

## 3. Chemical Composition, Polymerization, and Film Formation Mechanisms of Natural Lacquer

### 3.1. Chemical Composition of Natural Lacquer

Natural lacquer, also referred to as raw lacquer or Chinese lacquer, is a natural emulsion collected from the bark of Toxicodendron vernicifluum trees. It is not a single compound but a sophisticated non-aqueous microemulsion system, whose distinctive chemical composition gives it ultra-high durability, gloss, and chemical stability unmatched by most synthetic coatings [18]. The main components include urushiol, water, laccase, polysaccharides, glycoproteins, and minor trace ingredients [19].

Urushiol is the primary film-forming substance, accounting for 60–65 wt% of natural lacquer [20]. Chemically, urushiol belongs to a category of catechol derivatives with long aliphatic side chains. Its molecular structure consists of a benzene ring with two adjacent phenolic hydroxyl groups, linked to a C15 or C17 hydrocarbon chain containing various numbers of unsaturated double bonds [21]. The composition and saturation level of the side chain vary with lacquer tree species and growth region, directly affecting key coating properties such as curing rate, hardness, and flexibility [22].

Water makes up 20–30 wt% and acts as the continuous phase of the emulsion, which is essential for maintaining the catalytic activity of laccase and stabilizing the entire colloidal system [18]. Laccase, a copper-containing polyphenol oxidase present at approximately 0.2 wt%, serves as the biological catalyst that initiates the polymerization of urushiol [23]. In the presence of oxygen, laccase oxidizes the phenolic hydroxyl groups of urushiol to generate highly active radicals, triggering the subsequent cross-linking reactions [24].

Polysaccharides (3–7 wt%) and water-insoluble glycoproteins (1–2 wt%) function as emulsifiers and structural stabilizers [20]. Hydrophobic glycoprotein molecules self-assemble at the oil–water interface to form the outer layer of reverse micelles, encapsulating water, laccase, and metal ions into “micro-aqueous pools” [25]. This unique water-in-oil microstructure enables the stable coexistence and efficient reaction of urushiol, laccase, and water [26]. Trace components include stellacyanin, a blue copper protein present at about 0.02 wt%, which is believed to regulate the concentration of phenoxy radicals at the interface and control oxygen diffusion to sustain catalytic activity [27]. Metal ions such as Ca, Mg, and Cu also exist as cofactors for laccase or participate in stabilizing the colloidal network [28].

### 3.2. Polymerization Mechanism of Urushiol

The polymerization of natural lacquer is a complex cascade reaction initiated enzymatically and followed by free-radical oxidative polymerization, which can be divided into two main stages [24].

The first stage is the enzymatic initiation process, a biochemical reaction that determines the overall curing rate [18,24]. Under suitable temperature and high humidity, laccase catalyzes the oxidation of the catechol structure in urushiol [29]. With the assistance of oxygen, each urushiol molecule loses one electron and one proton to form a semiquinone radical with high reactivity. This step is widely recognized as the rate-limiting step of the entire film-forming process. Laccase contains a T1/T2/T3 copper cluster, and the four-electron transfer mechanism at the T1 copper site initiates the oxidation, followed by electron transfer to the T2/T3 trinuclear cluster where molecular oxygen is reduced to water [30,31].

Once urushiol radicals are generated, the reaction enters the second stage: non-enzymatic free-radical chain polymerization [18]. In this stage, urushiol radicals couple with each other mainly through C–C or C–O–C linkages to form dimers, which can be further oxidized to produce new radicals and continue propagating into oligomers [24]. Meanwhile, unsaturated double bonds on the long aliphatic side chains undergo auto-oxidation to form peroxides, which decompose into additional radicals. These side-chain reactions couple with the aromatic ring polymerization to promote the formation of a three-dimensional cross-linked network. Chain termination occurs primarily through radical combination or disproportionation, eventually leading to a highly cross-linked urushiol polymer network linked randomly by C–C and C–O–C bonds [24]. Temperature experiments showed that the activity of the four laccases (Nago-7, Nago-8, Nago-9, and Vietnam origin) increased initially with rising temperature and then decreased, each exhibiting an optimal reaction temperature. pH experiments revealed that the activity of all four laccases followed a bell-shaped curve within the tested pH range, each having its own optimal pH for reaction. Strain-specific differences in temperature and pH adaptability were observed among laccases from different sources. Laccase activity is highly sensitive to environmental pH and temperature [32], which directly determines the initiation rate of urushiol oxidation and the overall curing speed of raw lacquer. Controlling pH within 6.0–7.5 and temperature at 20–30 °C can maintain the highest catalytic efficiency, ensuring stable film-forming performance of collected lacquer sap [33].

### 3.3. Film Formation Process and Structure–Property Relationships

Film formation of natural lacquer involves not only chemical polymerization but also a series of physicochemical changes and supramolecular assembly processes. Recent studies have revealed that the lacquer emulsion contains a thin interfacial layer (approximately 2.43 nm) composed of urushiol, laccase–urushiol complexes, stellacyanin–urushiol complexes, and water-insoluble glycoproteins [19]. Radicals generated by laccase inside the micro-aqueous pools are efficiently transferred to the bulk urushiol phase through the glycoprotein-mediated interface, sustaining continuous polymerization [23,34].

As polymerization proceeds, high-molecular-weight urushiol polymers gradually accumulate and assemble with polysaccharides and glycoproteins via non-covalent interactions including hydrogen bonding, hydrophobic forces, and coordination bonds [35]. With the gradual evaporation of water, the system undergoes phase separation; the polymer concentration increases and solidifies into a dense cross-linked film [36]. In the final lacquer film, the polysaccharide–glycoprotein network acts like a reinforcing “skeleton” embedded in the urushiol polymer “matrix”, synergistically providing exceptional mechanical strength, thermal stability, solvent resistance, and long-term durability [37].

The structure–property relationship of natural lacquer is fundamentally determined by its composition and cross-linked architecture. Higher urushiol content and a higher degree of unsaturation in the side chains usually accelerate curing and improve hardness and gloss [38]. The stability of the emulsion and the uniformity of cross-linking directly influence film formation, surface quality, and adhesion [39]. The multi-component synergistic effect and the highly cross-linked supramolecular network endow natural lacquer with its signature long-term stability, aesthetic gloss, and outstanding protective performance, which form the mechanistic basis for subsequent modification, desensitization, nanocomposite preparation, UV curing, and the design of high-performance functional coatings.

## 4. Lacquer Tree Resources, Ecological Cultivation, and Raw Material Quality

### 4.1. Resource Distribution and Global Production Status

China is the world’s dominant producer and historical supplier of natural raw lacquer. According to the China Forestry Yearbook and national resource surveys, China’s annual raw lacquer production reached approximately 2500 tonnes in the early 2010s, with historical peaks exceeding 5000 tonnes in 2000 [40]. China has historically accounted for approximately 80% of international lacquer trade, underscoring its central role in the global supply chain [40]. The industry currently faces severe resource constraints: total lacquer tree resources have declined to less than 200 million trees, with artificial plantations comprising less than 10% of the total resource base [40]. The lacquer tree is distributed across 24 provinces in China, with cultivation and wild resources concentrated in the Qinba Mountains, Hengduan Mountains, Yunnan-Guizhou Plateau, and Sichuan Basin regions [41]. The lacquer tree forms the foundation of the lacquer industry, with its cultivation area, superior varieties, and harvesting techniques determining raw lacquer yield and quality. China has the largest lacquer resources and highest production globally, mainly in Shaanxi, Sichuan, Hubei, Yunnan, and Guizhou [42] (Table 1).

### 4.2. Varieties Characteristics of Major Lacquer Tree Varieties and Compositional Differences

China has a long history of lacquer cultivation, forming two major variety groups: large-wood and small-wood lacquer. They differ in physical characteristics, growth habits, raw lacquer properties, and ecological conditions, leading to distinct applications and development directions [43] (Table 2).

Large lacquer trees, also called “mountain” or “wild” lacquer trees, are mainly found in Yunnan, Guizhou, Sichuan, and Hunan. They are tall, slow-growing (30–40 years), with an economic life of ~10 years. Advantages include high-quality raw lacquer with high urushiol content, high viscosity, fast drying, and durable, lustrous, wear- and corrosion-resistant films suitable for high-end products. They are also highly adaptable to adverse environments, aiding soil and water conservation. However, industrialization is limited by long growth cycles, low yield per plant, and high tapping/processing costs.

### 4.3. Ecological Cultivation, Sustainable Tapping and Quality Regulation

**Figure 1:**
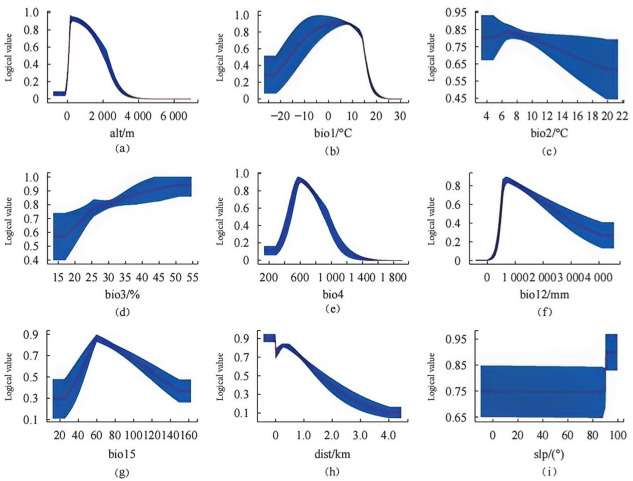
Response curves of lacquer tree distribution to nine environmental variables. (**a**) Response curve of Toxicodendron vernicifluum distribution suitability to altitude; (**b**) Response curve of Toxicodendron vernicifluum distribution suitability to annual mean temperature; (**c**) Response curve of Toxicodendron vernicifluum distribution suitability to maximum temperature of the warmest month; (**d**) Response curve of Toxicodendron vernicifluum distribution suitability to precipitation seasonality; (**e**) Response curve of Toxicodendron vernicifluum distribution suitability to temperature seasonality; (**f**) Response curve of Toxicodendron vernicifluum distribution suitability to annual precipitation; (**g**) Response curve of Toxicodendron vernicifluum distribution suitability to precipitation seasonality; (**h**) Response curve of Toxicodendron vernicifluum distribution suitability to distance to water bodies; (**i**) Response curve of Toxicodendron vernicifluum distribution suitability to slope [41].

Lacquer tree tapping is crucial for raw lacquer production. Traditional methods often involve over-tapping and rough operation, reducing tree growth, output, lifespan, and risking resource depletion. Establishing a scientific, sustainable tapping mechanism that balances yield and resource protection is essential. Sustainability is influenced by climate; habitat fluctuations challenge stability, and slight changes in temperature or precipitation can shift optimal growth zones (Figure 1) [41].

A climate-smart framework is recommended for macro-level resource planning [44]. Micro-level practices include standardizing the tapping period (June–September) and cycle: large trees every 5–7 days, small trees every 2–3 days, with 2–4 years recuperation. Continuous tapping and tapping during dormancy are prohibited [32].

**Figure 2:**
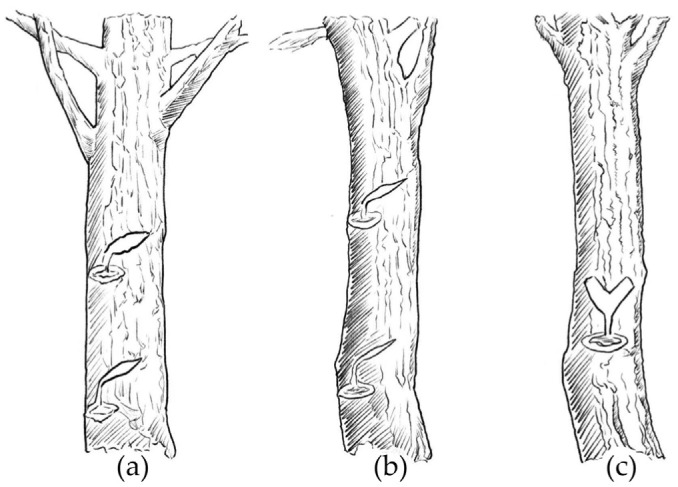
Tapping tool shapes and cutting methods: (**a**) Eyebrow-shaped blade; (**b**) Willow leaf-shaped blade; (**c**) Fish-tail-shaped blade.

Tapping methods and tools should be optimized. Traditional “one-cut” or excessively deep cuts damage phloem and xylem, hindering nutrient transport. Promote “oblique cutting” and “sectional tapping” with appropriate blade shapes: eyebrow (Figure 2a), willow leaf (Figure 2b), fish-tail (Figure 2c), controlling depth at 3/4 bark thickness. Modern tools like stainless-steel knives and special lacquer collectors reduce tree damage, improve efficiency, and increase recovery rate [45].

Post-tapping maintenance is essential for recovery and subsequent production. Wounds should be cleaned, disinfected, and protected to prevent infection, reduce loss, and accelerate callus formation. Whole-cycle management includes pruning, soil-based fertilization, and green pest control, promoting tree health and sustainable management.

## 5. Technological Innovation Drives Industrial Upgrading

Technological innovation is crucial for the lacquer industry, enabling solutions to existing problems, improving competitiveness, and achieving sustainable development. Currently, China’s lacquer production relies heavily on manual methods, with low output, unstable product quality, and insufficient R&D of new lacquer materials. Modernizing production processes and diversifying product types by integrating traditional crafts with new technologies is key to industrial upgrading.

### 5.1. Processing and Manufacturing Technologies

The production end covers raw lacquer tapping, collection, processing, and storage, where innovation focuses on improving efficiency, stabilizing quality, and reducing costs. Manual collection has long led to unstable raw material supply and inconsistent lacquer quality. 3D printing technology has been introduced into traditional lacquer craft production, enabling complex structural designs and reducing the technical barriers associated with conventional handcrafting methods [46]. Downstream processing has similarly benefited from automation. Automatic filtration, purification, dehydration, and refining systems with online quality detection improve primer consistency and reduce material waste. Automatic machines for filtering, mixing, and drying further streamline procedures and stabilize lacquer quality across production batches. In terms of storage and rheology management, inorganic salts such as Na_2_CO_3_ function as polymerization inhibitors, extending the non-crusting storage period to approximately two months and reducing material loss [47]. Fast and stable coagulants additionally improve rheological properties to meet the demands of modern mechanical application systems. Beyond the lacquer sap itself, modern industry increasingly emphasizes comprehensive utilization of by-products. Ultrasonic-assisted supercritical CO_2_ extraction of lacquer seed oil improves yield and purity [48], while dewaxing technology further enhances its commercial value [49]. This shift transforms traditional sap-focused production into a more complete resource utilization model, expanding product lines and enhancing overall industrial value.

### 5.2. Functional Modification of Lacquer-Based Polymer Materials

Natural lacquer exhibits inherent limitations including slow ambient curing, restricted color range, allergenicity, and moderate weathering resistance, all of which constrain its broader application. Molecular and composite modification strategies have therefore been extensively investigated to enhance its functional performance.

Allergen reduction represents a primary concern for widening the consumer base. Desensitization approaches include cyclodextrin/chitosan embedding, laccase-targeted mutation, antioxidant protective measures, terpene oxidation control, and nanofiber membrane filtration, each operating through distinct mechanisms to suppress urushiol sensitization (Table 3) [50].

**Figure 3:**
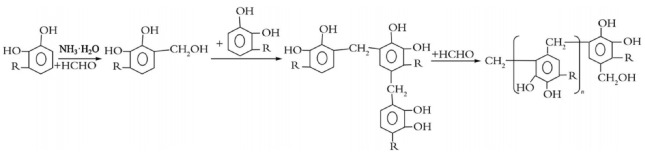
Synthesis of the lacquer phenol epoxy acrylate (LPEA) prepolymer [51].

To overcome the slow curing limitation, photopolymerization technology has been applied through the synthesis of urushiol-modified epoxy acrylate (LPEA) prepolymers (Figure 3). UV-curable LPEA systems reduce drying time from 3 h to 0.5 h and full curing time from 12 h to 2 h, while increasing impact resistance from 10 to 30 cm, enabling direct integration with modern UV spray-curing production lines [51].

Polyurethane hybrid systems represent another major modification route. By incorporating polyurethane segments into the lacquer network at varying ratios, the LPU series achieves a tunable balance between flexibility and hardness. Performance data show that LPU70 (lacquer/PU = 7:3) retains 88.3% gloss after 16 h UV irradiation and 54.9% gloss after 10 min water boiling, substantially outperforming unmodified lacquer [52]. With increasing PU ratio (0–50%), L rose from 6.42 to 53.43 and red-yellow chromaticity increased, indicating lighter, yellower coatings. Gloss retention after boiling and UV irradiation ranged 47.9–88.3% and 55.4–92.3%, respectively, both increasing with PU content; LPU50 performed best among blends (83.3%, 92.3%), though pure PU was highest (93.9%, 95.2%). Time-gloss curves showed continuous UV-induced decline, but LPU70 and above degraded markedly less than LPU100, confirming PU blending significantly improved water and UV resistance. The polyurethane segments introduce flexible aliphatic chains that reduce film brittleness, while urethane linkages provide additional hydrogen-bonding cross-links, collectively improving weathering and water resistance.

Nanomaterial incorporation further extends the functional range of lacquer-based coatings. Graphene oxide (GO) platelets dispersed within the urushiol matrix create a physical barrier that prolongs the diffusion path of corrosive media, significantly improving anticorrosion performance [53]. Nano-SiO_2_ fills microscopic defects in the cured film to enhance hardness and wear resistance, while cellulose nanocrystals serve as bio-based reinforcing agents that simultaneously improve mechanical strength and biocompatibility [54,55]. A comparative overview of the key modification strategies, including curing conditions and principal performance outcomes, is summarized in Table 4.

### 5.3. Applications and Technology Readiness

Lacquer-based polymer materials have expanded well beyond traditional decorative uses into a broad spectrum of functional applications, though their technology readiness levels vary considerably.

In functional protective coatings, lacquer-based systems with robust adhesion and weather resistance have been developed for automotive applications, providing durable surface protection and passive thermal management [56]. High-performance variants are also under investigation for aerospace, marine, and precision electronics applications. In the biomedical field, lacquer’s biocompatibility and antibacterial properties show potential for wound closure and antimicrobial coatings [57], though these remain largely at the laboratory stage. Soy protein-modified lacquer coatings have additionally been explored as sustainable building materials [58].

In environmental and separation applications, urushiol-based bio-superhydrophobic Janus membranes have demonstrated effective performance in seawater desalination and solar-driven evaporation systems, the preparation involves two steps: Fe(OH)_2_-coated cotton fabric is first obtained via FeSO_4_ immersion and ammonia treatment, then modified with urushiol solution to produce MCF. The resulting MCF, loaded with black urushiol-Fe microspheres, is placed in seawater under sunlight for solar-driven desalination [59], while lacquer-derived functional materials show promise for heavy metal adsorption and resource recovery [60]. These represent early-stage laboratory demonstrations rather than established industrial processes.

For cultural, decorative, and consumer applications, digital technologies including 3D modeling, CNC engraving, robotic spraying, and parametric design have enabled efficient production of complex lacquerware forms and personalized products. Collaboration with designers and cultural IP holders has further supported the development of lightweight cultural products such as jewelry, electronic accessories, and cosmetics derived from lacquer seed lipids [61], combining oriental aesthetics with contemporary design sensibility.

### 5.4. The Fourth and Fifth Industrial Revolutions and Their Application to the Lacquer Industry

The world is currently undergoing a profound transition from the Fourth Industrial Revolution (Industry 4.0) to the Fifth Industrial Revolution (Industry 5.0), a historical process that presents unprecedented technological opportunities and development frameworks for the modernization of China’s lacquer industry.

Industry 4.0 is driven by the Internet of Things (IoT), big data, artificial intelligence (AI), cloud computing, and automated robotics, and its essence lies in achieving a high degree of intelligent integration of physical and digital production systems [62]. In the lacquer industry, the influence of this revolution has already begun to emerge. As discussed in earlier sections, automated filtration, refinement, and online quality-detection systems have significantly improved batch-to-batch consistency of raw lacquer quality. Meanwhile, the introduction of 3D modeling, parametric design, and computer numerical control (CNC) engraving has made it possible to rapidly generate complex patterns and manufacture them with high precision, substantially shortening the cycle from creative design to finished product. However, for traditional lacquer craftsmanship, machines cannot fully replicate the tactile sensitivity and aesthetic judgment accumulated by a master artisan over decades; excessive automation risks eroding cultural meaning and breaking the transmission chain of traditional skills. It is in this context that the principles of Industry 5.0 offer a more integrative solution.

The core tenets of Industry 5.0 are human-centricity, sustainability, and resilience, emphasizing that technology should not replace human beings, but should work in symbiosis with human creativity [63]. In the lacquer industry, this philosophy implies the construction of a human–machine collaboration model. AI-assisted design systems can generate hundreds of design schemes based on traditional pattern databases within milliseconds, while the final aesthetic selection and refinement of craft details remain dependent on experienced lacquer artisans. Collaborative robots (cobots) can take over repetitive and time-consuming tasks such as lacquer-layer polishing and base-material blending, thereby freeing the artisan’s energy and attention for high-value activities such as creative expression and fine decorative work [64]. Furthermore, Industry 5.0’s emphasis on sustainability aligns closely with the goals of lacquer tree conservation and green production. At the product level, Life Cycle Assessment (LCA)-based green design tools can help optimize the material composition and production processes of lacquerware, reduce the emission of harmful substances, and respond to the growing international market demand for sustainable luxury goods [65].

## 6. Industrialization Models and Market Expansion

Industrialized operation is essential for the growth of the lacquer industry, serving as a bridge connecting raw material production, technological R&D, and consumer markets. Currently, China’s lacquer industry is in the early stage of industrialization, facing a short industrial chain, small enterprise scale, weak production–research integration, limited independent brands, and low social influence. To address this, a multi-subject, cooperative industrial development mechanism is required, integrating market planning, technology, and brand-driven value, guiding the industry from a resource-characteristic craft display model to a modern, value-oriented industrial system.

### 6.1. Industry-University-Research Integration Model: Strengthening Technical Support and Talent Training

The modern transformation of the lacquer industry depends on resolving technological bottlenecks and structural talent gaps. A deeply collaborative innovation ecosystem should integrate policy guidance, industrial demand, academic research, R&D strength, user feedback, and financial capital, with market demand as the guide and physical platforms as the hub [66]. Standardized physical innovation platforms, such as national or ministerial-level technology alliances or engineering research centers, can concentrate multidisciplinary strengths from universities, research institutes, and leading enterprises. Platforms should adopt mechanisms where industry proposes needs, the platform organizes implementation, collaborative teams develop solutions, and results are shared. Long-term joint research plans can focus on key technologies such as low-sensitization raw lacquer, high-quality composite coatings, intelligent equipment, green production, and integration of digital design and intelligent manufacturing [67]. Digital design frameworks, including 3D modeling and virtual simulation, can shorten development cycles, reduce prototyping costs, and enhance technological synergy.

Talent development is critical for industrialization. Universities and vocational colleges should establish interdisciplinary curricula combining lacquer art, materials science, chemical engineering, industrial design, intangible cultural heritage, and marketing to cultivate compound professionals who understand traditional culture, modern technology, market trends, and innovation. Digital tools and user experience design should be integrated to train professionals capable of narrating lacquer heritage and developing digital cultural products [68]. Joint construction of university-enterprise studios, involving masters, heritage inheritors, technical experts, and designers, can combine traditional craftsmanship with engineering technology. A two-way flow mechanism allows faculty to serve as enterprise consultants and project engineers, and enterprise personnel as industrial professors in universities, facilitating effective matching of knowledge supply and industry demand.

Sustainable collaboration requires benefit- and risk-sharing mechanisms. R&D investment and revenue models can pool government, enterprise, and social funds, stipulate IP ownership, and establish mixed-ownership industrial companies converting technology, capital, and labor into shares. Enterprises provide technical demands, pilot scale-up, and commercialization; universities and research institutes provide R&D and high-level talent training; governments provide guidance, support, and subsidies to reduce innovation risks. Customer information feedback platforms integrate market demand into R&D optimization and attract social capital as investors. This “industry-university-research” alliance evolves into a symbiotic, win-win partnership, ensuring continuous technological progress, industrial scaling, and sustainable market expansion.

### 6.2. Integration Model of Intangible Cultural Heritage Empowerment and Rural Revitalization: Broadening Development Paths and Social Benefits

The lacquer industry possesses rich intangible cultural heritage and rural resource endowments. As a national intangible cultural heritage, lacquer craftsmanship forms an important part of China’s traditional culture, while lacquer tree cultivation and tapping are concentrated in rural areas, aligning with national rural revitalization strategies. Constructing an integrated development model of “intangible cultural heritage empowerment + rural revitalization” can leverage innovative inheritance methods such as AI-assisted design and live e-commerce to promote cultural transmission, drive rural employment, and increase farmers’ income, achieving mutual promotion of industrial development and rural revitalization [69].

Digital technology is a key enabler for revitalizing intangible heritage and expanding value chains. Open and shared “Chinese Lacquer Pattern and Vessel Shape Gene Databases” combined with AI allow in-depth analysis and intelligent design of classic patterns and vessel shapes. For example, Tianjin Polytechnic University’s Visual Communication Design Department has digitized intangible heritage patterns to support product design, enabling rapid AI-assisted generation of design schemes and improving creative efficiency. Immersive technologies including 3D modeling, VR, and AR facilitate visual preview, user experience simulation, and personalized customization, while 3D printing and CNC engraving streamline production of complex lacquerware blanks and pre-carving, transforming craftsmanship from “experience-based” to “data-driven + skilled workmanship”. In the research on related technical fields, the MR (Mixed Reality) technology was mentioned the most (133 times), followed by VR (Virtual Reality, 35 times), AR (Augmented Reality) 11 times, 360-degree video 6 times, haptic feedback devices 5 times, and another 1 technology category [70]. High-precision digital archiving, motion capture, and multi-spectral imaging create “digital twin” records of endangered lacquer techniques and expert operations, supporting skill training and virtual teaching, expanding the inheritance base, and providing sustainable technical support [71,72].

**Figure 4:**
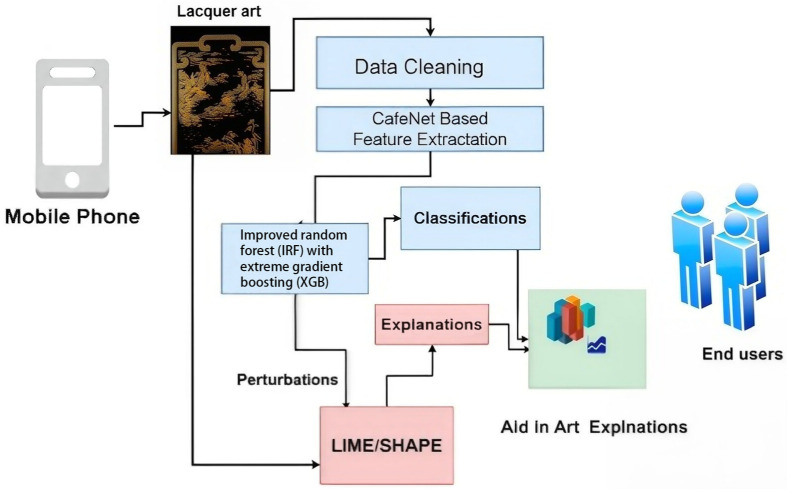
Overview of lacquer art explanation using LIME [71].

From a rural industrial perspective, the lacquer industry can promote agriculture-industry-tourism integration. Compound agricultural models such as lacquer tree intercropping with medicinal plants, fungi, or legumes improve land use, asset value, and ecological benefits. Experiential consumption through “workshop + cultural tourism” programs allows visitors to engage in lacquer production, from tree management and juice extraction to lacquer making and creation, staying in lacquer-themed accommodations, and purchasing original products. This transforms production spaces into cultural consumption and communication spaces, enhancing product premium, brand loyalty, and stimulating related service industries. E-commerce and online marketing expand sales channels beyond regional limits. Studies indicate that tourists’ awareness, immersive experience, and participation levels in regions such as Sichuan significantly influence consumption intentions and revisit behavior, highlighting the lacquer industry’s role as a culturally driven, experience-oriented sector [73] (Figure 4).

An innovative production and operation model integrating “leading enterprises + cooperatives + farmers + Internet” can ensure industrial sustainability. Governments guide companies, local talents, and cooperative organizations to standardize cultivation, tapping, grading, and pricing, guaranteeing farmers’ basic income. Cooperatives focus on primary processing, brand building, and live broadcast promotion, while enterprises handle deep processing, product development, brand management, and high-value sales. Information management ensures traceability of raw lacquer collection, processing, and sales. This model embeds small farmers into the industrial chain, distributes value-added benefits, and promotes risk-sharing and sustainable community development, fostering coordinated growth of cultural heritage, industry, and rural economy.

### 6.3. Market Education and International Communication: Enhancing Brand Influence and Cultural Value

To overcome cognitive barriers and the lack of brand recognition in the lacquer industry, systematic strategies from precise market education to brand building and international communication are essential. The goal is to reposition lacquer products from niche collectibles to high-end design materials and quality consumer goods, promoting lacquer culture globally and enhancing both cultural and economic value [74]. Against the backdrop of digitalization and cultural globalization, market expansion has shifted from traditional product export to an integrated model of cultural narrative, digital communication, and experience economy, constructing a universally understood expression system that transforms lacquer from a regional craft into a global cultural symbol.

Targeted market education should cater to the cognitive and acceptance levels of different consumer groups. For the mass market, messaging should emphasize “green, safe, and enduring” attributes of modern lacquer products, highlighting eco-friendly sourcing, low allergy risk, long service life, and simple maintenance. Public engagement through lifestyle influencers, product trials, derivative cooperation with popular home brands, and interior design media can strengthen recognition and appreciation. Digital tools, apps, and interactive displays enable participatory understanding of lacquer material, processing, and culture, enhancing conversion rates and brand loyalty. Designers should be positioned as users of high-end decorative materials, with access to professional forums, roadshows, and lacquer material libraries providing chromatograms, textures, and processing data to support interior and exterior design applications in high-end hotels, villas, art displays, and luxury brand stores [67].

**Figure 5:**
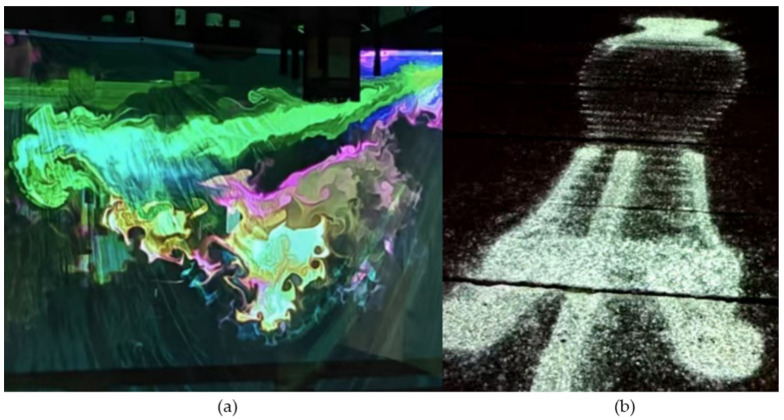
Interactive projection works: (**a**) Lacquer Flow Painting; (**b**) Luminous Treasure Vase [78].

Internationally, lacquer branding should emphasize its identity as a materialized form of oriental philosophy and contemporary artistic expression. High-level exhibitions, including the Venice Architecture Biennale, Milan Design Week, and Paris Deco Off, along with collaborations with foreign designers and institutions, transform the perception of lacquer from traditional craft to modern material art [75]. Digital and immersive experiences facilitate cultural understanding and engagement, moving from display-oriented to experience-driven communication. VR, AR, and interactive digital platforms allow global audiences to virtually participate in lacquer production, providing multisensory experiences and reducing geographic and temporal limitations (Figure 5a,b) [76,77,78].

A systematic brand and quality framework is necessary to ensure trust and international competitiveness. Dual-brand strategies—linking regional public brands with enterprise or designer brands—should be implemented with standardized certification systems. Regional brands, such as “Fuzhou bodiless lacquerware” and “Pingyao polished lacquerware,” should secure geographical indication certifications and enforce raw material, process, and quality standards. Enterprise and designer brands should focus on innovation, design, technological upgrading, and commercial operation, supported by 3D modeling and parametric design to digitally express and rapidly iterate products. A whole-industry quality and certification system covering lacquer tree varieties, raw lacquer grades, environmentally friendly additives, and product types ensures reliability and compliance with domestic and international standards, providing a “technical pass” and quality guarantee for global market access [79].

### 6.4. Global Manufacturing Landscapes: Challenges in Europe and the United States and Comparative Perspectives with China

While China remains the world’s dominant producer of natural lacquer and lacquerware, the material has found application outside Asia primarily through museum conservation, luxury goods integration, and specialist craft studios. In Germany, Kremer Pigmente GmbH (Munich) is among the few established European distributors supplying authentic urushi lacquer to conservators and craft practitioners [80]. In the United Kingdom and the United States, major institutions including the Victoria and Albert Museum, the Getty Conservation Institute, and the Museum of Fine Arts Boston maintain active lacquerware conservation programs [78].

Despite this growing international interest, the lacquer industry in Europe and the United States faces significant structural challenges. First, neither region cultivates lacquer trees domestically, creating complete dependency on Asian raw material imports and acute supply chain vulnerability [81]. Second, urushiol’s status as a potent allergen generates substantial regulatory pressure: the EU’s REACH Regulation subjects urushiol derivatives to SVHC scrutiny [82], while the American Academy of Dermatology estimates approximately 50 million Americans suffer from urushiol-induced contact dermatitis annually [83], creating commercial liability concerns.

A comparison of these regional challenges with China’s situation reveals both shared vulnerabilities and instructive asymmetries, as summarized in Table 5.

The comparison reveals that artisan scarcity and supply chain fragility are universally shared, though their causes differ: China’s risk is internal—declining cultivation incentives and an aging tapping workforce, whereas Europe and the United States lack any domestic production base entirely. This asymmetry reinforces the strategic importance of China’s resource development agenda, as a stable Chinese lacquer supply effectively functions as a global resource for the international lacquer community.

## 7. Conclusions and Suggestions

This review has examined the scientific foundations, technological innovations, and industrialization pathways of lacquer-based polymer materials, drawing on evidence spanning compositional chemistry, curing mechanisms, modification strategies, and application performance. Several conclusions of both scientific and practical significance emerge from this synthesis.

From a mechanistic standpoint, the curing behavior of natural lacquer is more sensitive to environmental variables than previously recognized in industrial practice. Laccase activity exhibits sharp optima with respect to both pH and temperature, and deviations from the range of pH 6.0–7.5 and 20–30 °C measurably impair cross-linking density and film uniformity. This sensitivity is rarely accounted for in processing specifications, representing a systematic gap between laboratory understanding and production reality. Similarly, while the glycoprotein-mediated interfacial structure has been identified as essential for sustaining radical transfer during polymerization, its deliberate manipulation as a processing variable remains largely unexplored.

Comparative analysis of modification strategies reveals a consistent pattern: performance gains in one dimension are frequently accompanied by trade-offs in others. Polyurethane hybridization improves weathering and flexibility but reduces the characteristic deep black tone valued in traditional lacquerware. Increasing GO loading enhances anticorrosion properties but introduces dispersion challenges that compromise film homogeneity at higher concentrations. UV-curable systems dramatically accelerate processing but require capital-intensive equipment incompatible with small-scale artisanal production. These trade-offs are insufficiently acknowledged in the existing literature, where individual studies tend to report optimized single-parameter outcomes without systematic cross-comparison. The absence of standardized testing protocols—covering substrate type, coating thickness, curing conditions, and aging methodology—further limits the interpretability and comparability of reported data across research groups.

Several important questions remain unresolved. The relationship between urushiol side-chain composition, which varies substantially across lacquer tree species and geographic origins, and the ultimate mechanical and barrier properties of cured films has not been quantitatively established. The long-term field durability of nanocomposite and hybrid lacquer coatings under real service conditions—particularly cyclic thermal stress, UV–visible irradiation, and biological exposure—has not been demonstrated beyond accelerated laboratory testing. In the domain of allergen reduction, no strategy reported to date has simultaneously achieved low sensitization, preserved film-forming performance, and demonstrated industrial scalability; these three requirements have only been addressed in isolation. Furthermore, biomedical and environmental applications, including cardiovascular coatings and Janus separation membranes, remain at early laboratory stages with no reported progression toward clinical or pilot-scale validation.

At the industrial level, the fragmentation of the lacquer value chain—separating resource cultivation, raw material processing, material modification, and product manufacturing into disconnected segments—constrains the translation of laboratory advances into commercial outcomes. The industry-university-research collaboration models discussed in this review offer a structural pathway toward integration, but their effectiveness depends on the establishment of shared performance standards, IP-sharing mechanisms, and sustained public funding that are currently inconsistent across regions.

In summary, lacquer-based polymer materials possess a scientifically grounded foundation for high-performance functional applications, but realizing this potential requires addressing three interconnected priorities: the development of standardized, cross-comparable performance evaluation frameworks; the systematic investigation of scalability and long-term stability for advanced modification systems; and the construction of integrated processing–modification–performance models that can guide rational material design. Progress on these fronts will determine whether the lacquer industry can transition from craft-based heritage production to a science-driven, sustainable materials platform with genuine global relevance.

## Figures and Tables

**Table 1:** Distribution of lacquer trees.

Species of Lacquer Trees	Distribution Map
Melanorrhoea usitata	Bhutan, Ho Chi Minh City
Rhus succedanea	Hanoi, Taiwan
Rhus vernicifera	Xi’an, Wuhan, Guiyang, Hirosaki

**Table 2:** Comparative morphological and physicochemical characteristics of large-wood lacquer and small-wood lacquer.

Characteristic	Large-Wood Lacquer	Small-Wood Lacquer
Source tree type	Tall arbor-type (Toxicodendron vernicifluum); wild or semi-wild cultivation	Shrub-type (Toxicodendron vernicifluum); intensively cultivated
Geographic distribution	Yunnan, Guizhou, Sichuan, Hunan	Shaanxi, Hubei, Sichuan, Guizhou, Chongqing
Tree morphology	Height 10–20 m; trunk diameter > 30 cm; slow growth (30–40 years to maturity)	Height 3–8 m; trunk diameter < 20 cm; fast growth (8–15 years to maturity)
Economic lifespan	~10 years of productive tapping	~15–20 years of productive tapping
Raw lacquer appearance	Light yellow to wheat yellow; high opacity	Deep yellow to purplish brown; relatively transparent
Viscosity	High; thick consistency	Moderate; relatively fluid
Stringing behavior (thread test)	Short threads; high elasticity and strong retraction	Long, slender threads; moderate elasticity
Particle dispersion (mi-yu test)	Dense, fine particles; readily form chain-like aggregates	Sparse, larger particles; slower aggregation
Odor	Mixed sour and sweet aroma	Fresh, mild aroma
Color change rate (oxidation)	Rapid; complete browning within ~20 min	Slow; complete browning within ~30 min
Sedimentation profile	Lower urushiol concentration in upper layer; higher in bottom layer	Higher urushiol concentration in upper layer; lower in bottom layer
Bottom aqueous phase (bone water)	Clear, lightly colored; <1% by volume	Yellowish-white, turbid; <5% by volume
Film-forming properties	Fast drying (high zaoxing); thick film; prone to wrinkling in storage	Slow drying (low zaoxing); thin film; stable during storage
Urushiol content	Lower (50–60 wt%)	Higher (60–70 wt%)
Laccase activity	Higher	Moderate
Film performance	Excellent hardness, gloss, and corrosion resistance; suitable for high-end applications	Good transparency and adhesion; suitable for general-purpose coating and artistic applications

**Table 3:** Strategy evaluation [50].

Mitigation Strategy	Key Experimental Support	Limitations	Industrial Challenges
Cyclodextrin/Chitosan Embedding	Encapsulates urushiol via hydrophobic cavities and spatial barriers.	No quantitative efficiency data; potential impact on film properties.	Compatibility with traditional recipes; process optimization needed.
Laccase Modification	Targeted mutations reduce quinone byproducts through altered catalytic pathways.	Complex genetic engineering and microbial expression requirements.	High production costs; acceptance in traditional craftsmanship.
Antioxidant Protective Equipment	Antioxidant additives (e.g., vitamin C) inhibit free radical formation.	Short-lived efficacy; limited protection against airborne VOCs.	Disposable equipment costs; worker compliance with PPE.
Terpene Oxidation Control	Antioxidants (e.g., propyl gallate) delay oxidation; controlled humidity and temperature reduce reaction rates.	Requires energy-intensive environmental control; potential storage stability issues.	Retrofitting production lines; additive–resin compatibility.
Nanofiber Membrane Filtration	High-surface-area membranes adsorb and filter VOCs for PPE and ventilation.	Clogging risk with high VOC loads; balance between breathability and efficiency.	Scalable manufacturing costs; long-term material durability.

**Table 4:** Comparative overview of key modification strategies for lacquer-based polymer materials.

Modification Strategy	Key Agent	Curing Conditions	Principal Performance Outcome	Limitation
Desensitization (embedding)	Cyclodextrin/Chitosan	Ambient	Reduced allergenicity; broader consumer applicability	Potential impact on film properties; process optimization needed
Desensitization (enzymatic)	Laccase mutation	Ambient	Suppressed quinone byproduct formation	High production cost; complex genetic engineering
UV-curable derivative	LPEA prepolymer	UV, ~0.5 h	Drying time (decline) 83%; curing time (decline) 83%; impact resistance (rise) 200% (10→30 cm)	Equipment investment for UV spray-curing lines
Polyurethane hybrid	Lacquer/PU = 7:3 (LPU70)	UV, 16 h	Gloss retention 88.3% (UV); 54.9% (boiling water)	Reduced blackness; color shift at high PU ratios
Graphene oxide nanocomposite	GO platelets	Ambient	Significantly improved anticorrosion and mechanical properties	Dispersion uniformity; scalability
Nano-SiO_2_/CNC incorporation	Nano-SiO_2_; cellulose nanocrystals	Ambient	Enhanced hardness, wear resistance, and biocompatibility	Long-term stability under UV exposure not fully established

**Table 5:** Comparative overview of key challenges in the lacquer industry across China, Europe, and the United States.

Challenge Dimension	China	Europe	United States
Raw material supply	Domestic; declining cultivation area	Fully import-dependent	Fully import-dependent
Allergen/regulatory pressure	Emerging; desensitization R&D underway	REACH/SVHC scrutiny	High liability; public sensitization risk
Artisan talent pipeline	Formal programs exist; insufficient scale	Near-absent; informal only	Minimal practitioner base
Cultural market recognition	Growing but low public awareness	Perceived as foreign/niche	Confused with synthetic lacquer
Industrialization level	Early-stage; fragmented production	Non-industrial; conservation-focused	Non-industrial; museum and luxury only
Key competitive advantage	Scale, biodiversity, R&D momentum	Conservation expertise; luxury brand access	Advanced materials research

## Data Availability

No new data were created or analyzed in this study. Data sharing is not applicable to this article.
